# The relationship between the antibiotic consumption in hospitals and the community and the selection of methicillin-resistant *Staphylococcus aureus*

**DOI:** 10.1186/1753-6561-5-S6-O72

**Published:** 2011-06-29

**Authors:** L Kardaś-Słoma, PY Boëlle, D Guillemot, L Temime

**Affiliations:** 1 Pierre and Marie Curie University, Paris, France; 2 Inserm, U 707, Paris, France; 3 CNAM, Paris, France; 4 Institut Pasteur,PhEMI, Paris, France

## Introduction / objectives

The impact of reductions in antibiotic use on the selection of antibiotic-resistant bacteria such as methicillin-resistant *Staphylococcus aureus* (MRSA) is difficult to assess, all the more so as they are often accompanied by a concomitant increase in the use of a specific antibiotic class.

## Methods

We developed a computerized model of *S. aureus* transmission in a hospital ward and in the community, where methicillin-sensitive (MSSA), community-associated MRSA (CA-MRSA) and hospital-associated MRSA (HA-MRSA) strains co-circulate between these two settings. We assessed the impact of a 15-50% reduction in antibiotic use in community and hospitals, under several hypotheses regarding changes in the distribution of prescribed antibiotics.

## Results

An overall reduction in antibiotic use in the community led to decreased frequency of MRSA but increased carriage of *S. aureus* in both hospital and community settings. A similar reduction in hospitals had no impact on community dynamics.

Antibiotic classes-specific changes over the time period of the reduction in global ambulatory antibiotic use had an important impact on MRSA selection in hospitals and in the community. For instance, in the hypothesis of a 15% antibiotic use reduction, the induced decrease in hospital MRSA frequency was 2.6 times less important if this was accompanied by a concomitant 30% increase in the use of quinolones.

## Conclusion

Antibiotic reduction policies may not prove sufficient to decrease antibiotic-resistance frequency. They should include a surveillance of changes in the consumption of each antibiotic class.

## Disclosure of interest

None declared.

